# Can Aidi injection alleviate the toxicity and improve the clinical efficacy of radiotherapy in lung cancer? A meta-analysis of 16 randomized controlled trials following the PRISMA guidelines

**DOI:** 10.1097/MD.0000000000004517

**Published:** 2016-09-02

**Authors:** Zheng Xiao, Rui Liang, Cheng-qiong Wang, Shaofeng Xu, Nana Li, Yuejuan He, Fushan Tang, Ling Chen, Hu Ma

**Affiliations:** a Evidence-Based Medicine Center, MOE Virtual Research Center of Evidence-based Medicine at Zunyi Medical College, Affiliated Hospital of Zunyi Medical College; b Department of Respiratory Medicine (Center for Evidence-Based and Translational Medicine of Major Infectious Diseases), Affiliated Hospital of Zunyi Medical College; c Grade 2012 Students, Department of Public Health; d Department of Clinical Pharmacy, Zunyi Medical College; e Department of Oncology, Affiliated Hospital of Zunyi Medical College, Zunyi, Guizhou, China.

**Keywords:** Aidi injection, attenuation and synergistic efficacy, lung cancer, meta-analysis, radiotherapy

## Abstract

**Background/Introduction:**

Aidi injection plus radiotherapy is widely used for lung cancer in China. Can Aidi injection alleviate the toxicity and improve the clinical efficacy of radiotherapy in lung cancer? Has Aidi injection the attenuation and synergistic efficacy to radiotherapy? There is lack of strong evidence to prove it.

**Objectives:**

To reveal its real attenuation and synergistic efficacy to radiotherapy and provide sufficient evidence for adjuvant chemotherapy strategies to lung cancer, we systematically evaluated all related studies.

**Data Sources:**

We collected all studies about Aidi injection plus radiotherapy for lung cancer in Medline, Embase, Web of Science, China national knowledge infrastructure database (CNKI), Chinese scientific journals full-text database (VIP), Wanfang database, China biological medicine database (CBM) (established to June 2015), and Cochrane Central Register of Controlled Trials (June 2015), evaluated their quality according to the Cochrane evaluation handbook of randomized controlled trials (5.1.0), extracted data following the PICO principles and synthesized the data by Meta analysis.

**Results:**

Sixteen randomized controlled trials (RCTs) with 1192 lung cancer patients were included, with general methodological quality in most trials. The merged relative risk (RR) values and their 95% CI of meta-analysis for objective response rate (ORR), disease control rate (DCR), and quality of life (QOL) were as follows: 1.54, (1.39,1.70), 1.10 (1.02, 1.19), and 2.13 (1.68, 2.68). The merged RR values and their 95% CI of meta-analysis for myelosuppression and neutropenia, radiation pneumonitis, and radiation esophagitis were as follows: 0.51 (0.38, 0.69), 0.53 (0.42, 0.65), 0.52 (0.41, 0.67), and 0.52 (0.40, 0.68). All were statistically significant. The possibility of publication bias was small which objectively reported the results.

**Conclusions:**

The evidence available indicates that Aidi injection plus radiotherapy can significantly improve the clinical efficacy and QOL of patients with lung cancer. Aidi injection can alleviate the myelosuppression, radiation pneumonitis, and radiation esophagitis of radiotherapy. It has the attenuation and synergistic efficacy to radiotherapy.

## Abstract 2

Supplemental Digital Content is available in the text

## 1 Introduction

Lung cancer is the leading cause of cancer-related mortality in all countries with a 5-year survival rate of only 15%.^[1,2]^ Nonsmall cell lung carcinoma (NSCLC) accounts for approximately 80% of patients with primary lung cancers and over 50%of patients with NSCLC are advanced local invasion and distantmetastasis.^[3]^ Most patients are forced to accept chemotherapy, radiotherapy, or immunotherapy because of losing the chance of surgery.^[4,5]^ Radiotherapy and radiochemotherapy are important therapies for lung cancer.^[6,7]^ But their clinical efficacy is often limited by the radiotherapy-related toxicity including the myelosuppression, radiation pneumonitis, and radiation esophagitis that seriously reduce the patient's survival quality and prognosis.^[8–10]^ Therefore, it is the key problem how to alleviate the toxicity and improve the clinical efficacy.

Aidi injection (Z52020236, China Food and Drug Administration (CFDA)) is an adjuvant chemotherapy drug commonly used in China, which is composed of the extracts from Astragalus, Eleutherococcus senticosus, Ginseng, and Cantharidin. Astragalus, Eleutherococcus senticosus, Cantharidin and Ginseng, and others are important traditional Chinese medicine, which appear to have antitumor activity, immunoregulation, and attenuation to the acute or subacute toxicity induced by chemotherapy.^[11–16]^ Our previous meta-analysis (to be published) showed that Aidi injection has the attenuation and synergistic efficacy to chemotherapy through reducing acute or subacute toxicity, controlling or killing malignant cells in NSCLC. Aidi injection plus radiotherapy is an important treatment strategy for lung cancer commonly used in China. Can Aidi injection alleviate the toxicity and improve the clinical efficacy of radiotherapy in lung cancer? Has Aidi injection the attenuation and synergistic efficacy to radiotherapy? Related studies^[17–21]^ had shown that Aidi injection might alleviate the toxicity and improve the clinical efficacy of radiotherapy in lung cancer. These conclusions were different in different studies with lower sample size. There is lack of strong evidence to prove it. To reveal its real attenuation and synergistic efficacy to radiotherapy and provide sufficient evidence for adjuvant treatment strategies to lung cancer, we systematically evaluated all related studies.

## 2 Methods

This article followed Preferred Reporting Items for Systematic Reviews and Meta-Analyses (PRISMA) guidelines.

### 2.1 Literature search strategy

Two reviewers (C-qW and NL) independently searched articles in electronic databases using the search strategy (“Lung Neoplasms” [Mesh] OR Pulmonary Neoplasms OR Lung Neoplasm OR Pulmonary Neoplasm OR Lung Cancer OR Lung Cancers OR Pulmonary Cancer OR Pulmonary Cancers OR lung carcinoma OR Pulmonary carcinoma OR NSCLC) AND (aidi OR aidi injection). The databases were Medline, Embase, Web of Science, China National Knowledge Infrastructure Database (CNKI), Chinese Scientific Journals Full-Text Database (VIP), Wanfang Database, China Biological Medicine Database (CBM) (established to June 2015) and Cochrane Central Register of Controlled Trials (Issue 6 of 12, June 2015). All retrievals were implemented by the Mesh and free word. No language restrictions were placed on the search. Ethical approval was not required, as our study is a meta-analysis of published studies.

### 2.2 Studies inclusion and exclusion criteria

#### 2.2.1 Inclusion criteria

Included studies must meet the following criteria: The disease was diagnosed and confirmed with lung cancer by histopathological and cytological diagnostic criteria which pathological and clinical types were not limited. There were randomized controlled trials (RCTs) groups. The experimental group was Aidi injection plus radiotherapy and control group was radiotherapy which types were not limited. Subjects before being included in the study were not using other anticancer drugs of Chinese herbs. Outcomes. Clinical efficacy: according to the World Health Organization (WHO) guidelines^[22]^ for solid tumor responses, the indicators of the short-term efficacy were complete response (CR), partial response (PR), no change (NC), progressive disease (PD), objective response rate (ORR) equals CR + PR and disease control rate (DCR) equals CR + PR + NC. The short-term efficacy was evaluated by ORR and DCR. Quality of life (QOL): according to KPS grading system, the life quality was considered to be improved if KPS score was 10 points higher after the treatment. Attenuation: according to WHO standards^[22]^ for anticancer drug acute/subacute toxicity (0–4 grading system), the attenuation was evaluated by hematotoxicity (myelosuppression and neutropenia), radiation pneumonitis, and radiation esophagitis.

#### 2.2.2 Exclusion criteria

Excluded studies must meet the following criteria: duplicated articles. Unrelated studies including studies of other themes, animal studies and in vitro studies. Nonrandomized controlled study studies. Meeting abstracts and reviews without specific data. All studies that report information are not accurate and statistical data cannot be used.

### 2.3 Study quality evaluation

We evaluated the quality of all included studies according to the Cochrane evaluation handbook of RCTs (5.3).^[23]^ The bias parameters were the random sequence generation (selection bias), allocation concealment (selection bias), the blinding of participants and personnel (performance bias), blinding of outcome assessment (detection bias), incomplete outcome data (attrition bias), selective reporting (reporting bias), and the other bias. We judged each item on three levels (“Yes” for low bias, “No” for a high risk of bias, and “Unclear”). Then, we assessed the trials and categorized them into three levels: low risk of bias (all the items were categorized “Yes”), high risk of bias (at least 1 item ranked “No”), and unclear risk of bias (at least 1 item was “Unclear”).

### 2.4 Selection and evaluation of articles

Two reviewers (RL and SX) independently selected and evaluated articles according to the above standards. Any disagreements were resolved by discussion with each other or with ZX.

### 2.5 Data extraction and statistical analysis

Two reviewers (RL and YH) independently extracted all data including: publishing time and country. Study design overview including the randomization methods, demographic characteristics, and blinding implementation. The sample size of experimental and control group, the clinical efficacy, QOL, and attenuation. Meta-analysis was done by 2 reviewers (C-qW and SX) with Review Manager 5.3 (The Cochrane Collaboration, Oxford, UK). The relative risk (RR) and 95% confidence intervals (CI) were calculated. Statistical heterogeneity of the results across trials was assessed by *χ*^2^ based Q-statistic test and the inconsistency was calculated by *I*^2^. If the homogeneity (*P* ≥0.1, *I*^2^ ≤50%) was not rejected, the fixed-effects model was used to calculate the summary relative risk (RR) and the 95% CI. The results were analyzed by random-effects model if the homogeneity (*P* <0.1, *I*^2^ >50%) was higher and the results of the fixed and random effect model had good consistency. The clinical heterogeneity was reduced by strict inclusion and exclusion criteria and subgroups analysis. Statistical heterogeneity was reduced by random effects model if the results of the fixed and random effect model had good consistency. Otherwise, the results were analyzed by descriptive analysis. Publication bias was evaluated through funnel plots if the included studies were more than 10.

## 3 Results

### 3.1 Search results

**Figure 1:**
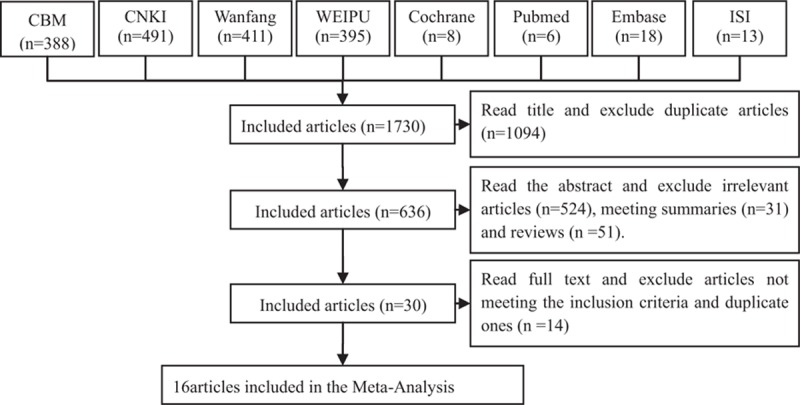
Articles retrieved and assessed for eligibility. After successively applying the study exclusion criteria, 16 RCTs were included. RCTs = randomized controlled trials.

The initial database search identified 1730 articles using our search strategies (Fig. 1). After successively applying the study exclusion criteria, 16 RCTs were included.

### 3.2 Characteristics of the included studies

There were 16 RCTs with 1192 lung cancer patients being included in this meta-analysis (Table 1).^[17–21,24–34]^ The cases of Aidi injection plus radiotherapy and radiotherapy were 597 and 595 respectively. The male and female were 800 and 392 respectively which age was between 26 and 90. The dosage of Aidi injection was 50 to 100 mL/times. The time and course were different in different institutions. Clinical efficacy, QOL, and attenuation were reported.

**Table 1:**
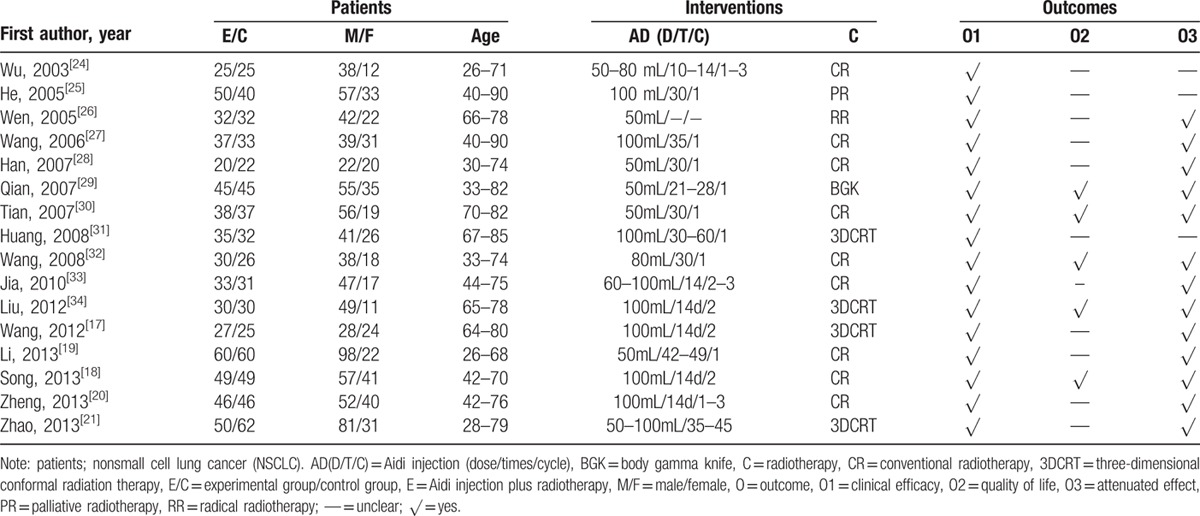
Characteristics of the included studies.

### 3.3 Methodological bias of the included studies

**Figure 2:**
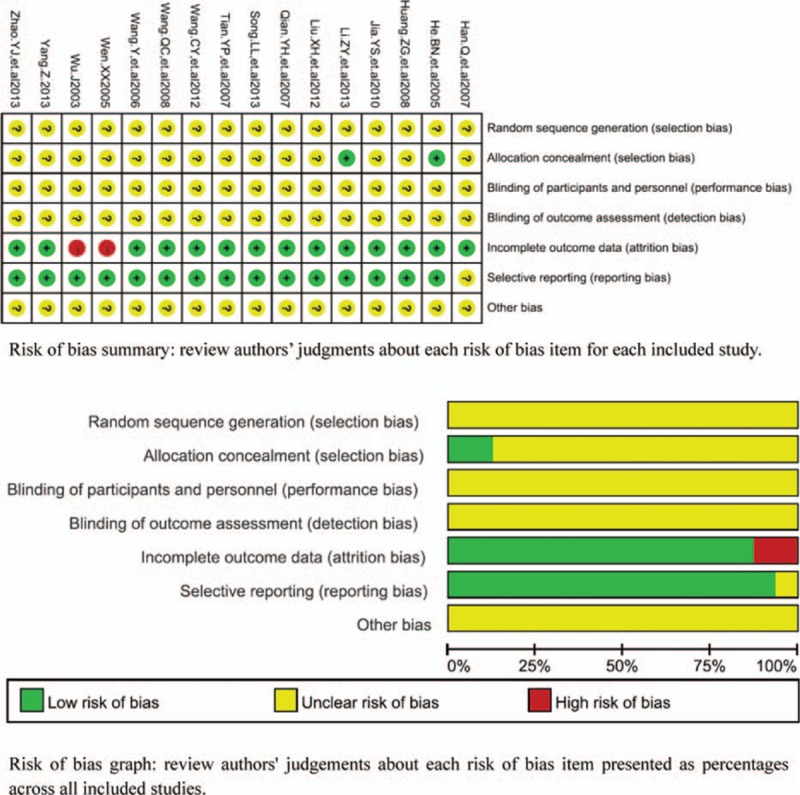
Risk of methodological bias of the included studies. There was general methodological quality in most trials.

All 16 trials did not describe the methods of random allocation which indicated that there was a possibility of high selectivity bias in the included studies. In 2 trials, the random allocation concealment was implemented by envelope. In others, the random allocation concealment was not described. The blinding to patients and doctors was not described in all studies. These indicated that there were the selective bias and high implementation bias. In 2 trials, missing data was not processed by intention to treat analysis (ITT) and data were complete in other trials. There was not the selective reporting in all studies. Other bias was not clear. Characteristics and quality of all included studies are presented in Fig. 2.

### 3.4 Clinical efficacy

According to the WHO guidelines for solid tumor responses, the clinical efficacy was evaluated by objective response rate (ORR) and disease control rate (DCR).

**Figure 3:**
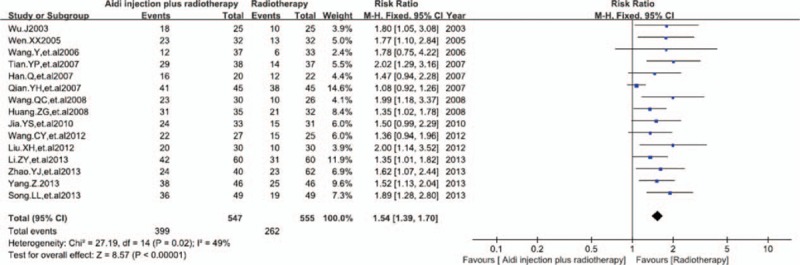
Meta-analysis of the ORR between 2 groups. Aidi injection plus radiotherapy could significantly improve the ORR of patients with lung cancer. ORR = objective response rate.

In 16 RCTs, 15 trials with 1102 cases were included (Fig. 3). There was lower statistical heterogeneity between studies according to the heterogeneity test (*I*^2^ = 49%). Meta-analysis showed that the ORR was statistically different between the 2 groups [RR = 1.54, 95% CI (1.39, 1.70), *P* <0.00001] by fixed-effects model. This showed that to compare with radiotherapy alone, Aidi injection plus radiotherapy could significantly improve the ORR of patients with lung cancer.

**Figure 4:**
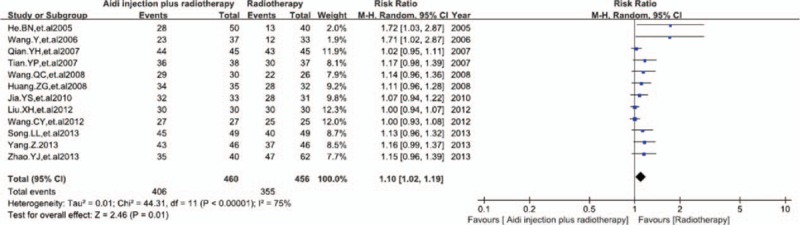
Meta-analysis of the DCR between 2 groups. Aidi injection plus radiotherapy could significantly improve the DCR of patients with lung cancer. DCR = disease control rate.

Twelve trials with 916 cases were included (Fig. 4). There was higher statistical heterogeneity between studies (*I*^2^ = 75%). Meta-analysis showed that the DCR was statistically different between the 2 groups [RR = 1.10, 95% CI (1.02, 1.19), *P* = 0.01] by random effect model. This showed that Aidi injection plus radiotherapy could significantly improve the DCR of patients with lung cancer.

### 3.5 Quality of life (QOL)

**Figure 5:**
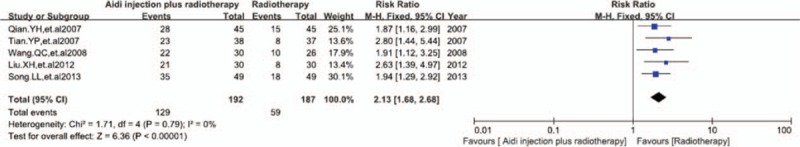
Meta-analysis of the QOL improvement rate between 2 groups. Aidi injection plus radiotherapy could significantly improve the QOL of patients with lung cancer. QOL = quality of life.

According to Karnofsky standard, the quality of life was evaluated by KPS score. In 16 RCTs, 5 trials with 379 cases were included (Fig. 5). There was no statistical heterogeneity between studies (*I*^2^ = 0%). Meta-analysis showed that the QOL was statistically different between the 2 groups [RR = 2.13, 95% CI (1.68, 2.68), *P* <0.00001] by fixed-effects model. This showed that Aidi injection plus radiotherapy could significantly improve the QOL of patients with lung cancer.

### 3.6 Attenuation

**Figure 6:**
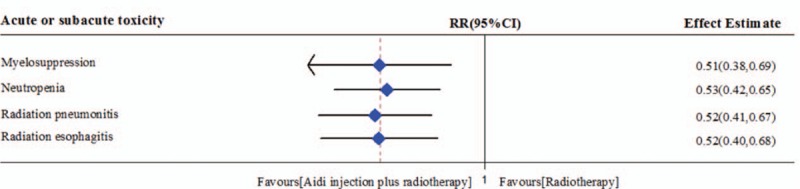
Meta analysis results of acute or subacute toxicity between 2 groups. Aidi injection can alleviate the myelosuppression, radiation pneumonitis, and radiation esophagitis of radiotherapy.

According to the WHO standards,^[22]^ the acute or subacute toxicity was evaluated by hematotoxicity (myelosuppression and neutropenia), radiation pneumonitis and radiation esophagitis (Table 2 and Fig. 6).

**Table 2:**
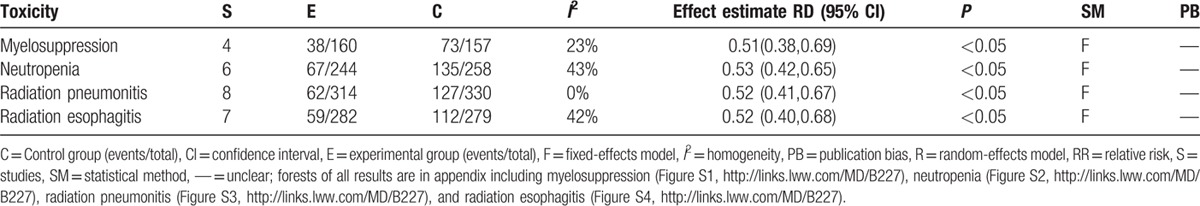
Meta analysis results of acute or subacute toxicity between 2 groups.

### 3.7 Myelosuppression

In 16 RCTs, 4 trials with 317 cases were included (Table 2, Fig. 6 and Figure S1). There was lower statistical heterogeneity between studies (*I*^2^ = 23%). Meta-analysis showed that the myelosuppression rate was statistically different between the 2 groups [RR = 0.51, 95% CI (0.38, 0.69), *P* <0.00001] by fixed-effects model. This showed that Aidi injection plus radiotherapy could significantly reduce the incidence rate of myelosuppression of patients with lung cancer.

### 3.8 Neutropenia

In 16 RCTs, 6 trials with 502 cases were included (Table 2, Fig. 6 and Figure S2). There was lower statistical heterogeneity between studies (*I*^2^ = 43%). Meta-analysis showed that the neutropenia rate was statistically different between the 2 groups [RR = 0.53, 95% CI (0.42, 0.65), *P* <0.00001] by fixed-effects model. This showed that Aidi injection plus radiotherapy could significantly reduce the neutropenia of patients with lung cancer.

### 3.9 Radiation pneumonitis

In 16 RCTs, 8 trials with 644 cases were included (Table 2, Fig. 6 and Figure S3). There was no statistical heterogeneity between studies (*I*^2^ = 0%). Meta-analysis showed that the radiation pneumonitis rate was statistically different between the 2 groups [RR = 0.52, 95% CI (0.41, 0.67), *P* <0.00001] by fixed-effects model. This showed that Aidi injection plus radiotherapy could significantly reduce the radiation pneumonitis of patients with lung cancer.

### 3.10 Radiation esophagitis

In 16 RCTs, 7 trials with 644 cases were included (Table 2, Fig. 6 and Figure S4). There was lower statistical heterogeneity between studies (*I*^2^ = 42%). Meta-analysis showed that the radiation esophagitis rate was statistically different between the 2 groups [RR = 0.52, 95% CI (0.40, 0.68), *P* <0.00001] by fixed-effects model. This showed that Aidi injection plus radiotherapy could significantly reduce the radiation esophagitis of patients with lung cancer.

### 3.11 Publication bias and heterogeneity analysis

#### 3.11.1 Publication bias analysis

**Figure 7:**
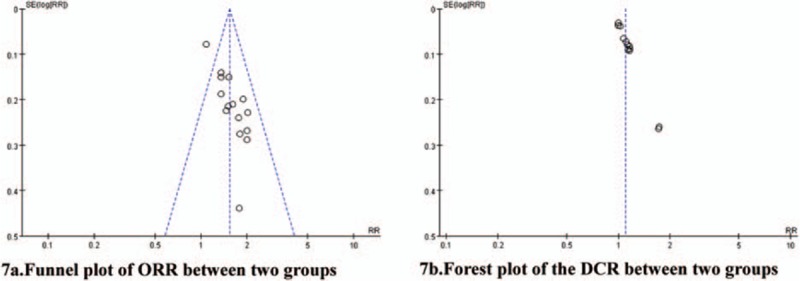
Publication bias analysis. There was lower publication bias in this study.

Fig. 7A was the funnel plot based on studies with data on ORR. Result showed that all points in the funnel plots were asymmetric and 1 point was distributed beyond the funnel. This indicated that there might be publication bias in our study that influenced the results of our analysis. Fig. 7B was the funnel plot based on studies with data on DCR. Results showed that all points in the funnel plots were asymmetric. This indicated that there was lower publication bias in this study. After excluding the over or underestimate studies, meta-analysis showed that the results before and after exclusion had a good consistency.

#### 3.11.2 Heterogeneity analysis

There was higher statistical heterogeneity between studies according to the heterogeneity test (*χ*^2^ = 44.31, *P* < 0.00001, *I*^2^ = 75%) in meta-analysis of DCR. The results between the fixed effects model and random effect model had a good consistency. So the meta-analysis is reliable.

## 4 Discussion

In this study, 16 RCTs with 1192 lung cancer patients were finally included. To compare with radiotherapy alone, meta-analysis showed that Aidi injection plus radiotherapy could significantly improve the ORR and DCR of patients with lung cancer. There was lower publication bias and the results may be underestimated or overestimated. After excluding the over or underestimate studies, meta-analysis showed that the results before and after exclusion had a good consistency. QOL was evaluated by KPS score according to Karnofsky standard. Meta-analysis showed that Aidi injection plus radiotherapy could significantly increase KPS score and improve the QOL without publication bias. These showed that Aidi injection plus radiotherapy could significantly improve increase the clinical efficacy and QOF of patients with lung cancer. Sodium cantharidinate is the semi-synthetic derivative of cantharidin. In vitro studies^[35]^ showed that cantharidin could inhibit the tumor cells proliferation or induce the tumor cells apoptosis. Animal studies^[35,36]^ also showed that cantharidin could significantly inhibit the liver cancer cells growth, enhance the immune function, and prolong the median survival time of tumor-bearing mice. Many studies^[11–16]^ also show that Astragalus, *Eleutherococcus senticosus*, Cantharidin, and Ginseng have antitumor activity and immune regulation functions. These studies indirectly confirmed that Aidi injection could increase the clinical efficacy through combating cancer and enhancing the immune function. In this meta-analysis, the subgroup analysis was not performed according to different radiotherapy or different pathological types. Therefore, it was unclear whether the clinical efficacy has difference in Aidi injection with different radiotherapy or lung cancer with different pathological types. So far, there was no reliable evidence to confirm the long-term effect. This needs to be confirmed by new strong evidence. In summary, to compare with radiotherapy alone, Aidi injection plus radiotherapy could significantly improve the ORR and DCR of patients with lung cancer. These studies reveal that Aidi injection has synergistic efficacy to radiotherapy in lung cancer.

In 16 RCTs, the radiotherapy-related toxicity including myelosuppression, radiation pneumonitis, and radiation esophagitis was reported according to the WHO standards.^[22]^ Many clinical or animal experimental studies^[37–40]^ showed that Chinese medicinal herbs could alleviate the radiotherapy-related toxicity. Aidi injection is an adjuvant chemotherapy drug commonly used in China. Can Aidi injection alleviate the toxicity of radiotherapy in lung cancer? This meta analysis showed that Aidi injection plus radiotherapy could significantly reduce the incidence rate of myelosuppression, neutropenia, radiation pneumonitis, and radiation esophagitis in patients with lung cancer. The results have good objectivity. Therefore, all results showed that Aidi injection could alleviate the radiotherapy related toxicity in lung cancer. Other similar meta-analysis^[41,42]^ showed that Aidi could decrease the incidence of radiation esophagitis and myelosuppression in esophageal carcinoma.^[43]^ The animal experimental study also showed that Aidi injection could prevent and treat the radiation-induced lung injury.^[44]^ This provides indirect evidence for the above outcomes. These studies show that Aidi injection can alleviate the myelosuppression, neutropenia, radiation pneumonitis, and radiation esophagitis in lung cancer. Aidi injection has the attenuation to radiotherapy in NSCLC.

## 5 Limitations

There were more limitations in this study. First, the included studies were published in Chinese which may be language bias. Second, all 16 trials did not report the random allocation method. The random allocation concealment was implemented in 2 trials. The blinding to patients and doctors were not described in all studies. These indicated that there were the selective bias and implementation bias and lead to overestimate the efficacy of the treatment group. Third, all included trials did not reported follow-up and the long-term efficacy. This might lead to an inadequate assessment to the clinical efficacy. Fourth, there was publication bias in included studies. In all, the evidence from this study is insufficient and needs to be further confirmed by standardized studies including large sample RCT or real-world studies.

## 6 Conclusion

This meta-analysis suggests that Aidi injection plus radiotherapy can significantly improve the clinical efficacy and QOL of patients with lung cancer. Aidi injection can alleviate the myelosuppression, radiation pneumonitis, and radiation esophagitis of radiotherapy. It has the attenuation and synergistic efficacy to radiotherapy. The quality of included studies is inadequate. Its attenuation and synergistic efficacy need to be confirmed by further large sample RCT or real-world studies with longer follow-up.

## Supplementary Material

Supplemental Digital Content
