# Supplementary material for: Can Aidi injection alleviate the toxicity and improve the clinical efficacy of radiotherapy in lung cancer? A meta-analysis of 16 randomized controlled trials following the PRISMA guidelines
Source: Medicine (Baltimore). 2016 Sep 2;95(35):e4517. doi: 10.1097/MD.0000000000004517 (PMC5008545; doi:10.1097/MD.0000000000004517)
Supplement: Supplemental Digital Content [file medi-95-e4517-s001.doc]

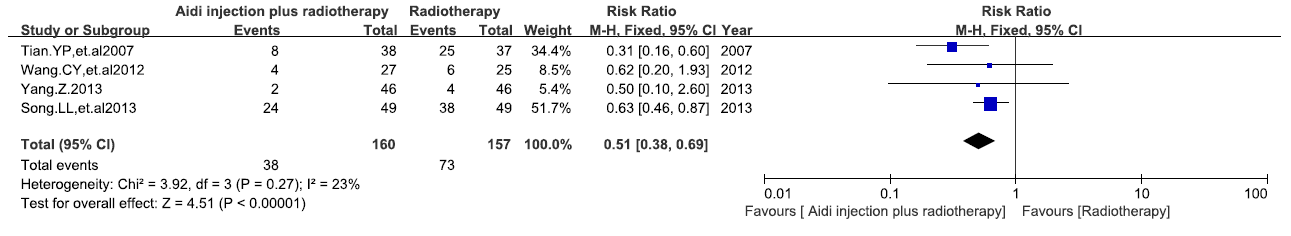


**Figure S1.Meta-analysis of the myelosuppression rate between two groups**


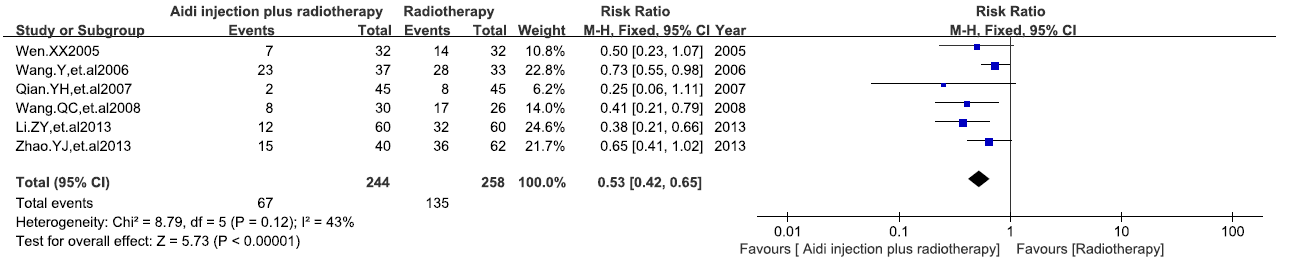


**Figure S2.Meta-analysis of the neutropenia rate between two groups**

**
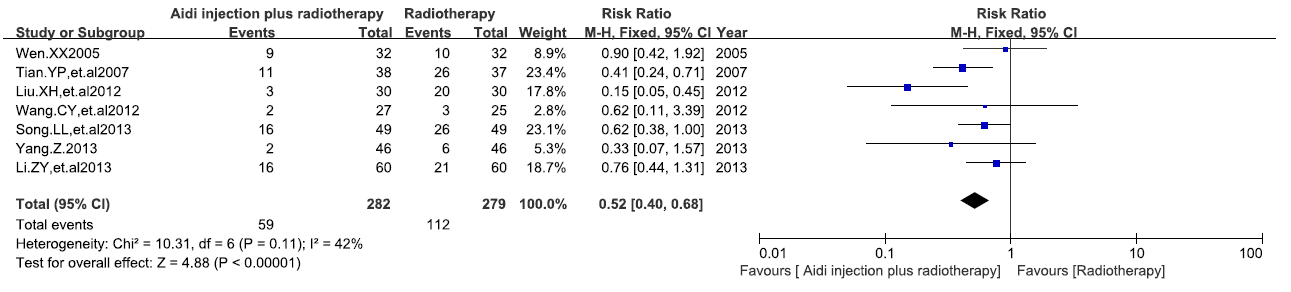
**

**Figure S3 Meta-analysis of the radiation pneumonitis rate between two groups**


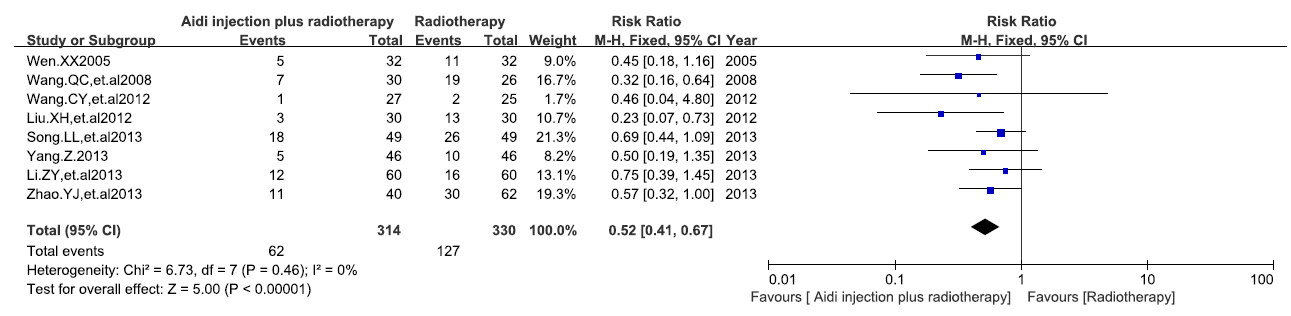


**Figure S4.Meta-analysis of the radiation esophagitis rate between two groups**
